# Expression of novel fusion antiviral proteins ricin a chain-pokeweed antiviral proteins (RTA-PAPs) in *Escherichia coli* and their inhibition of protein synthesis and of hepatitis B virus in vitro

**DOI:** 10.1186/s12896-018-0458-6

**Published:** 2018-08-06

**Authors:** Yasser Hassan, Sherry Ogg, Hui Ge

**Affiliations:** 1 Ophiuchus Medicine Inc., 1800 - 510 West Georgia Street, Vancouver, BC V6B 0M3 Canada; 2 Johns Hopkins University, AAP, Baltimore, MD 21218 USA; 3 AscentGene Inc., Gaithersburg, MD 20878 USA

**Keywords:** Fusion proteins, Ricin, Pokeweed antiviral protein, Hepatitis B virus, Antiviral agent, Ribosome-inactivating proteins

## Abstract

**Background:**

Ricin A chain (RTA) and Pokeweed antiviral proteins (PAPs) are plant-derived N-glycosidase ribosomal-inactivating proteins (RIPs) isolated from *Ricinus communis* and *Phytolacca Americana* respectively. This study was to investigate the potential production amenability and sub-toxic antiviral value of novel fusion proteins between RTA and PAPs (RTA-PAPs). In brief, RTA-Pokeweed antiviral protein isoform 1 from seeds (RTA-PAPS1) was produced in an *E. coli* in vivo expression system, purified from inclusion bodies using gel filtration chromatography and protein synthesis inhibitory activity assayed by comparison to the production of a control protein Luciferase. The antiviral activity of the RTA-PAPS1 against Hepatitis B virus (HBV) in HepAD38 cells was then determined using a dose response assay by quantifying supernatant HBV DNA compared to control virus infected HepAD38 cells. The cytotoxicity in HepAD38 cells was determined by measuring cell viability using a tetrazolium dye uptake assay. The fusion protein was further optimized using in silico tools, produced in an *E. coli* in vivo expression system, purified by a three-step process from soluble lysate and confirmed in a protein synthesis inhibition activity assay.

**Results:**

Results showed that RTA-PAPS1 could effectively be recovered and purified from inclusion bodies. The refolded protein was bioactive with a 50% protein synthesis inhibitory concentration (IC50) of 0.06 nM (3.63 ng/ml). The results also showed that RTA-PAPS1 had a synergetic activity against HBV with a half-maximal response concentration value (EC50) of 0.03 nM (1.82 ng/ml) and a therapeutic index of > 21,818 with noticeable steric hindrance. Results also showed that the optimized protein ricin A chain mutant-Pokeweed antiviral protein isoform 1 from leaves (RTAM-PAP1) could be recovered and purified from soluble lysates with gain of function on protein synthesis inhibition activity, with an IC50 of 0.03 nM (1.82 ng/ml), and with minimal, if any, steric hindrance.

**Conclusions:**

Collectively, our results demonstrate that RTA-PAPs are amenable to effective production and purification in native form, possess significant gain of function on protein synthesis inhibition and anti-HBV activities in vitro with a high therapeutic index and, thus, merit further development as potential potent antiviral agents against chronic HBV infection to be used as a standalone or in combination with existent therapies.

## Background

Pokeweed antiviral proteins (PAPs) are expressed in several organs of the plant pokeweed (*Phytolacca Americana)* and are potent type I Ribosome Inactivating Proteins (RIPs). Their sizes vary from 29-kDa to 30-kDa and are able to inhibit translation by catalytically removing specific adenine residues from the large rRNA of the 60S subunit of eukaryotic ribosomes [1–3]. Furthermore, PAPs can depurinate specific guanine residues, in addition to adenine, from the rRNA of prokaryotic ribosomes. PAPs possess antiviral activity on a wide range of plant and human viruses through various mechanisms [1]. Transgenic plants expressing different forms of PAPs were found to be resistant to various viral and fungal infections [4, 5]. The anti-viral activity of PAPs against human viruses has been described against Japanese encephalitis virus [6], human immunodeficiency virus-1 (HIV-1) [7], human T-cell leukemia virus-1 (HTLV-1) [8], herpes simplex virus (HSV) [9], influenza [10], hepatitis B virus (HBV) [11], and poliovirus [12]. PAPs low to moderate cytotoxicity to non-infected cells, in contrast to infected cells, makes PAPs very attractive candidates in the development of potential therapeutics and as protective agents against pathogens in transgenic plants.

Ricin is expressed in the seeds of the castor oil plant (*Ricinus communis*) and is one of the most potent type II RIPs. It is highly toxic to mammalian cells as its A chain can efficiently be delivered into the cytosol of cells through the mechanism of its B chain. The B chain serves as a galactose/N-acetylgalactosamine binding domain (lectin) and is linked to the A chain via disulfide bonds [13]. Ricin can induce 50% apoptosis in mammalian cells at concentrations below 1 ng/mL while showing no to low activity on plant and *E. coli* ribosomes. It is important to note however that the ricin A chain (RTA) on its own has less than 0.01% of the toxicity of the native protein in a cell culture test system. It was furthermore shown that RTA alone had no activity on non-infected and tobacco mosaic virus (TMV)-infected tobacco protoplasts alike. RTA lacks the ability to enter the cell without the action of the B chain [14]. RTA depurinates a universally conserved adenine residue within the sarcin/ricin loop (SRL) of the 28S rRNA to inhibit protein synthesis. Though there are currently no commercially available therapeutic applications, RTA is extensively studied in the development of immunotoxins [15].

The therapeutic potential of PAPs and RTA has been explored for over thirty years, though dosage dependant side effects have limited clinical applications. These proteins have shown very low cytotoxicity to non-infected cells; however, PAPs administration in mouse models has resulted in hepatic, renal and gastrointestinal tract damage with a median lethal dose (LD50) as low as 1.6 mg/Kg [16]. Interestingly, RTA shows no toxicity even at high doses with similar half-life times. Nevertheless, all RIPs show immunosuppressive effects to various degrees. Many studies have described the various dose-limiting side effects of these proteins when used as immunotoxins (i.e. vascular leak syndrome, hemolytic uremic syndrome and pluritis, among others) [17, 18]. Nonetheless, some patients achieved complete or partial remission against Refractory B-Lineage Acute Lymphoblastic Leukemia with sub-toxic dosages, for example.

Fusion and hybrid proteins of RTA and PAPs have also been developed in pursuit of selectively targeting infected cells and selectively recognizing viral components, though with limited success [19, 20]. Indeed, the engineering of novel therapeutic fusion proteins with higher specificity, selectivity, and potency with fewer side effects is a leading strategy in drug development that is more often than not limited by our still new understanding of protein structure and function. Another limiting factor is the availability of efficient protein structure prediction and simulation software. It is only with the recent advent of more sophisticated in silico tools that protein engineering became a viable alternative to conventional drug discovery techniques.

Based on the data gathered on these two proteins over the last thirty years and the newly available in silico tools, the authors hypothesized that it is possible to create novel fusion proteins between RTA and PAPs that will be more effective than either of the proteins alone at sub-toxic dosages against specific infectious diseases and that will be cheaper to produce than available therapeutics. The purpose of this research is thus to assess the potential production amenability and sub-toxic antiviral activities of newly created and biologically active novel fusion proteins between RTA and PAPs [21, 22].

Here, we describe the development of an effective and scalable production system in *Escherichia coli* and of purification methods that enabled accurate determination of RTA-PAPs protein synthesis inhibition in vitro. We similarly describe the in vitro reduced cytotoxicity and significant anti-HBV activity of RTA-Pokeweed antiviral protein isoform 1 from seeds (RTA-PAPS1) by detecting HBV DNA in the supernatant of HepAD38 cells. The reengineering of RTA-PAPS1 into RTA mutant- Pokeweed antiviral protein isoform 1 from leaves (RTAM-PAP1) to improve its production in *Escherichia coli* and to enhance its gain of function is also described using the most up-to-date protein structure and function prediction software available online.

## Methods

### *E. coli* in vivo expression system and rabbit reticulate lysate protein synthesis inhibition

#### Design of the DNA sequences of the proteins for *E. coli* in vivo expression system

The two cDNA sequences coding for RTA-PAPS1 (541 amino acids) and for RTAM-PAP1 (556 amino acids including the N terminal 6-His tag) were optimized for *E. coli* expression and chemically synthesized by AscentGene.

#### *E. coli* in vivo expression vector

The cDNA coding for RTA-PAPS1 and RTAM-PAP1 sequences described above were generated by PCR using the primers RP1-A48 (5’TTTAACTTTAAGAAGGAGATATA**CATATG**ATCTTCCCGAAACAGTACC) or RPAP1-A48 (5’TTTAACTTTAAGAAGGAGATATA**CATATG**CACCACCATCACCACCATA) and RPAP1-B50 (5’CAGCCGGATCTCAGTGGTGGTG**CTCGAG**TTAGGTAGTCTGGCAAGAACCG). Each PCR fragment was then subcloned into the *E. coli* pET30a expression vector (Novagene) between the NdeI and XhoI restriction endonuclease sites to generate the pET30a-RP1 and pET30a-6H-RPAP1 vectors respectively. The inserts were validated by DNA sequencing.

#### *E. coli* in vivo protein production

The above described vectors were transformed into *E. coli* BL21(DE3) cells (NEB) and expression of the proteins were examined from individual clones and analyzed by either Western blot using a monoclonal antibody specific to ricin A chain (ThermoFisher, RA999) or SDS gel stained with Comassie blue (ThermoFisher). Optimal conditions were determined and protein production induced in the presence of 1 mM IPTG from 1 L culture for each protein. The bacteria were then harvested by centrifugation, followed by lysing the cell pellets with 50 ml of lysis buffer (50 mM Tris-Cl, 150 mM NaCl, 0.2% Triton X100 and 0.5 mM EDTA). After sonication (3x2min), the soluble lysates were recovered by centrifugation at 35 K rpm for 40 min. The insoluble pellets were further extracted with 40 ml of 6 M Urea and the inclusion bodies (IB) were recovered by centrifugation at 16 K rpm for 20 min. Clarified IB were then dissolved with 20 ml of buffer 8b (proprietary formulation of AscentGene). The soluble proteins were then recovered by centrifugation (please contact the authors for more details).

#### *E. coli* protein purification

Ricin-PAPS1 proteins were purified by gel filtration column (Superdex 200 from GE Healthcare) under denaturing condition (6 M Urea). Peak fractions were pooled and powder Guanidine was added to a concentration of 5 M for complete denaturing. Denatured Ricin-PAPS1 was then added dropwise to the refolding buffer (50 mM Tris-Cl, pH 8.1, 0.4 M L-Arginine, 0.5 mM oxidized glutathione and 5 mM reduced glutathione) for refolding. The solution was stirred at room temperature for 10 min before allowing the refolding reaction to be further carried out at 4 °C for > 20 h. Clarified and refolded Ricin-PAPS1 proteins were then concentrated before going through the endotoxin removal process and the ammonium sulfate precipitation step. The resulting mixture was dialyzed in the formulation buffer containing 20 mM HEPES-Na, pH 7.9, 20% glycerol, 100 mM NaCl, 2.5 mM tris(2-carboxyethyl)phosphine (TCEP) and 1 mM EDTA.

The purification of the native RTAM-PAP1 from soluble lysate was achieved by affinity versus His-tag on Ni-sepharose column (GE Healthcare). After extensive washes with the lysis buffer, loosely bound proteins were eluted with the lysis buffer containing 40 mM Imidazole (I40). RTAM-PAP1 proteins were eluted with the elution buffer (20 mM Tris-Cl, pH 7.9, 100 mM NaCl, 1 mM EDTA and 300 mM Imidazole). A second purification step using Hydroxylapatite column (GE Healthcare) was used to further separate RTAM-PAP1 from co-purified host proteins. A third purification step, gel filtration on a fast protein liquid chromatography (FPLC) column of Superose 12 (GE Healthcare), was necessary to completely get rid of degraded and/or premature protein products. The resulting mixture was dialyzed in the formulation buffer containing 20 mM HEPES-Na, pH 7.9, 200 mM NaCl, 0.2 mM CaCl2 and 0.5 mM EDTA.

#### Rabbit reticulate lysate protein synthesis inhibition

The inhibitory activities of RTA-PAPS1 and RTAM-PAP1 were tested by using the Rabbit Reticulate Lysate TnT® Quick Coupled Transcription/Translation System and the Luciferase Assay System (Promega). Briefly, each transcription/translation reaction was performed according to the instructions for use (IFU) in the presence of a T7 Luciferase reporter DNA, and the Luciferase expression level was determined with a Wallac Microplate Reader. Transcription/translation runs were done twice with and without addition of five different concentrations of RTA-PAPS1 and RTAM-PAP1 in order to determine the inhibitory effect of the proteins. RTA-PAPS1 and RTAM-PAP1 concentrations were adjusted by taking sample purity into consideration.

### Anti-HBV assay

The anti-HBV assay was performed as previously described [23] with the modification of using HepAD38 cells by ImQuest BioSciences. ImQuest BioSciences developed a multi-marker screening assay utilizing the HepAD38 cells to detect proteins, RNA, and DNA intermediates characteristic of HBV replication. The HepAD38 cells are derived from HepG2 stably transfected with a single cDNA copy of hepatitis B virus pregenomic RNA, in which HBV replication is regulated by tetracycline. Briefly, HepAD38 cells were plated in 96-well flat bottom plates at 1.5 × 10^4^ cells/well in Dulbecco’s modified Eagle’s medium supplemented with 2% FBS, 380 μg/mL G418, 2.0 mM L-glutamine, 100 units/mL penicillin, 100 μg/mL streptomycin, and 0.1 mM nonessential amino acids (ThermoFisher). After 24 h, six ten fold serial dilutions of RTA-PAPS1 prepared in the same medium were added in triplicate. Lamivudine (3TC from Sigma Aldrich) was used as the positive control, while media alone was added to cells as a negative control (virus control, VC). Three days later, the culture medium was replaced with fresh medium containing the appropriately diluted RTA-PAPS1. Six days following the initial administration of RTA-PAPS1, the cell culture supernatant was collected, diluted in qPCR dilution buffer, and then used in a real-time quantitative qPCR assay using a Bio-Rad CFX384 Touch Real-Time PCR Detection System. The HBV DNA copy number in each sample was interpolated from the standard curve by the supporting software. A tetrazolium dye uptake assay (ThermoFisher) was then employed to measure cell viability, which was used to calculate cytotoxic concentration (TC50).

### Protein design optimization

#### Physiochemical profiling and specific structural features

The molecular profile of the protein was determined using the Protparam tool of ExPASy [24], and the solubility of these proteins was determined using Predict Protein [25]. The presence of disulfide bonds was determined using the DiANNA 1.1 webserver [26–28]. Functional effects of point mutations were determined using SNAP2 of Predict Protein.

#### Structure modeling

The structure of the protein was predicted by fold recognition methodology using the I-TASSER [29–31] and Phyre2 [32] prediction servers. The determined protein structures were then validated by Verify 3D [33, 34]. The quality of the structure was determined using the QMEAN6 program of the SWISS-MODEL [35] workspace.

#### Design of RTAM-PAP1

Three major changes were made to RTA-PAPS1 in order to increase its solubility, its efficacy against infected cells and to further reduce its toxicity.

Firstly, two point mutations, as predicted by SNAP2 of Predict Protein to have the least effect on function, were introduced into the RTA moiety to replace the Cysteine (Cys) residues with Alanine residues in order to completely avoid unwanted disulfide bond formation at position 171 and 259 (C171A and C259A) to create RTA mutant (RTAM).

Secondly, the natural semi-flexible linker previously used was replaced with a newly designed soluble flexible G rich linker with a rigid CASP2 recognition site (GGGGSDVADI(GGGGS)_2_) to allow better autonomous function of each moiety with minimal steric hindrance and to further enhance the chimeric protein’s ability to induce cell apoptosis [36].

Thirdly, A different variant than PAPS1 was used, PAP1, retrieved from National Centre for Biotechnology Information database (NCBI) with access number **P10297.2** in order to further enhance activity against HBV and further reduce toxicity of the chimeric protein.

Lastly, a 6-His tag was added at the N terminal of the protein RTAM-PAP1 in order to minimize effect on structure and function and to increase native protein recovery from *E. coli* production.

## Results

### Production and purification of recombinant RTA-PAPS1 in *E. coli* culture

**Fig. 1:**
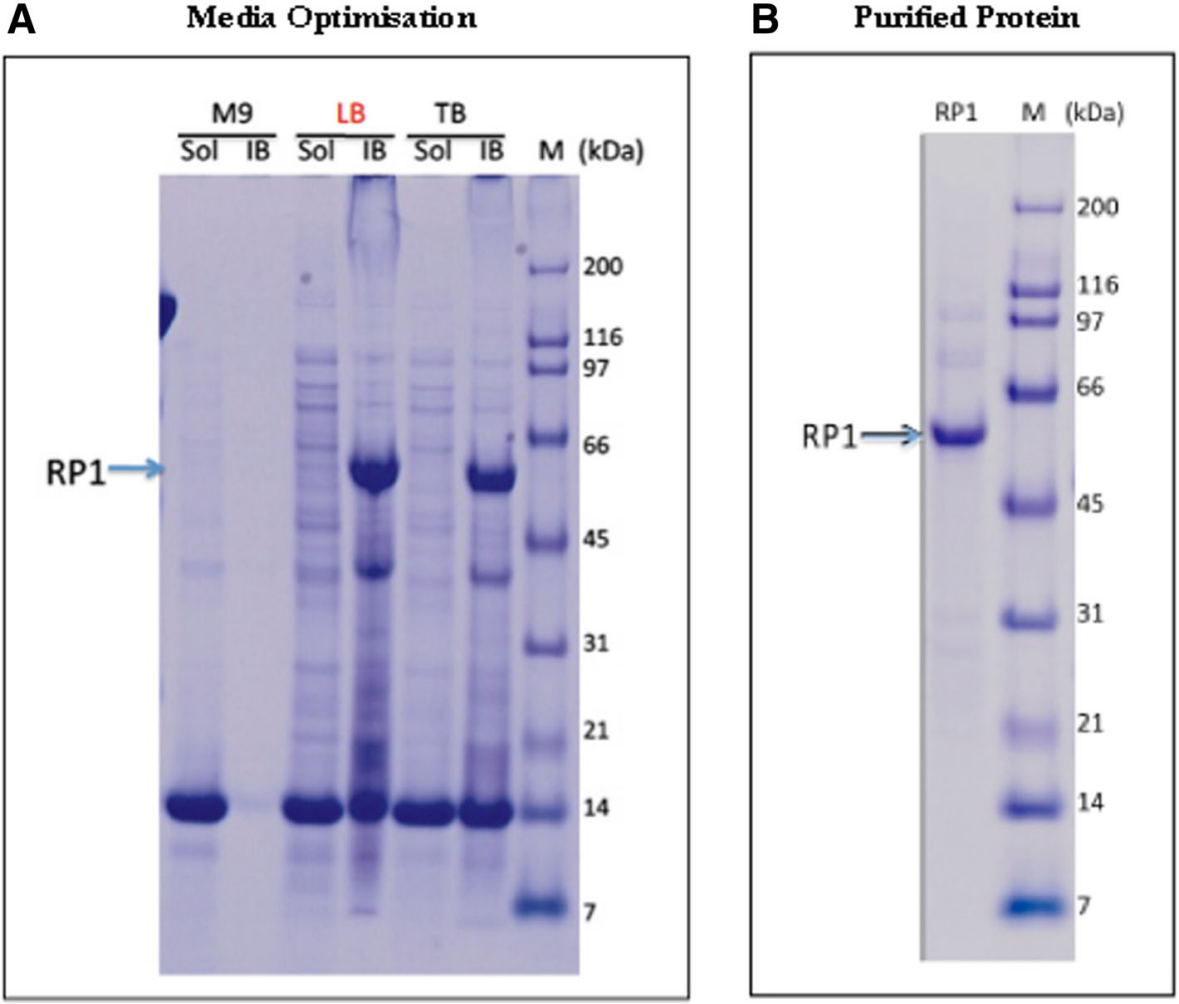
Medium Optimization and Protein Purification. **a** Medium optimization for Ricin-PAPS1 (RP1) expression. Three different growth media including M9 (M9), Luria Bertani (LB) and terrific broth (TB) were tested for Ricin-PAPS1 expression at 30 °C. Soluble lysate (Sol) and inclusion body (IB) from each sample were analyzed by SDS PAGE and visualized by Coomassie blue staining. **b** Validation of purified Ricin-PAPS1 protein. Recombinant Ricin-PAPS1 was produced in 1 L of culture that was induced with the optimized condition (LB medium with 1 mM IPTG at 30 °C for 4 h) and purified from inclusion bodies through gel filtration before refolding, concentration and dialysis. The resulting protein of approx. 60.5 kDa was > 90% purity determined by SDS-PAGE

The production of fusion Ricin A Chain-Pokeweed Antiviral Protein from Seeds Isoform 1 (RTA-PAPS1) in *E. coli* was found to be significantly better at 30 °C than at 37 °C (results not shown). In order to optimize the amount of protein produced from 1 L at 30 °C, three media were tested: M9 (M9), Luria Bertani (LB) and terrific broth (TB). Soluble lysate (Sol) and inclusion body (IB) from each sample were analyzed by SDS PAGE and visualized by Coomassie blue staining (Fig. 1a). As can be seen, almost all of the overexpressed RTA-PAPS1 proteins were in the form of inclusion bodies, which were almost completely insoluble in either 6 M Urea or 6 M Guanidine. A total of 28 proprietary buffers were tested and only the denaturing buffer 8b (proprietary formulation of AscentGene) was able to dissolve more than 50% of the Ricin-PAPS1 present in the inclusion bodies. Once the soluble proteins were recovered and purified through the gel filtration column Superdex200 (single step) in their denatured form, they were allowed to refold for over 20 h in a refolding buffer before being concentrated. The resulting protein was found to be at a concentration of 0.22 mg/ml at > 90% purity (Fig. 1b), which was the best result we obtained thus far after testing numerous production methods and bacterial strains, as previously described [21, 22].

### Inhibitory activity of recombinant RTA-PAPS1 in the rabbit reticulate lysate TnT® system

**Fig. 2:**
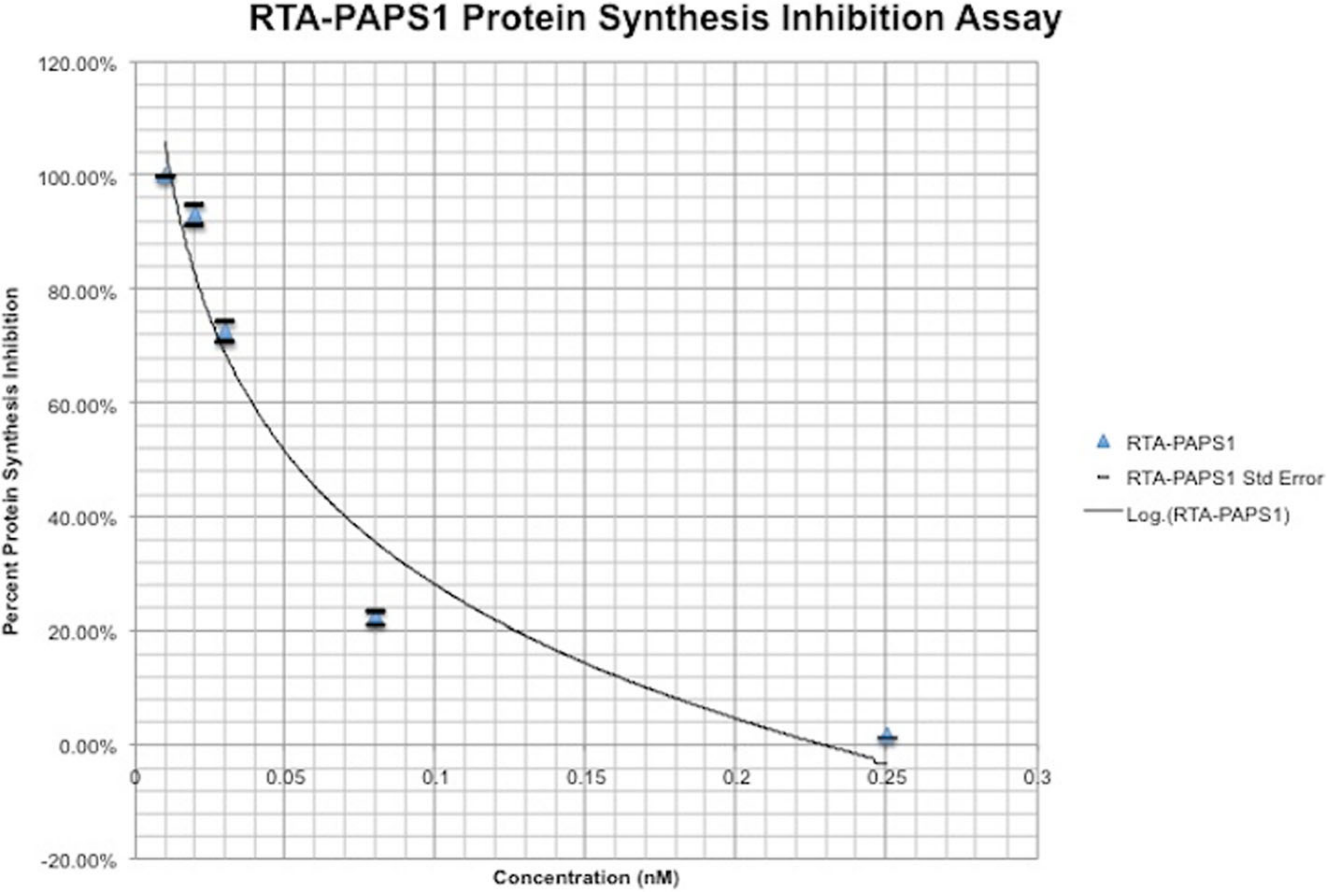
Test of purified RTA-PAPS1 in the TnT transcription/translation assay. Five different concentration points (0.01 nM, 0.02 nM, 0.03 nM, 0.08 nM, 0.25 nM) were examined. Values are calculated as percent of Luciferase protein synthesis compared to control. Results represent the mean for two individual experiments and the curve is the logarithmic regression

The inhibitory activity of RTA-PAPS1 was determined using 5 different concentrations of purified RTA-PAPS1 in duplicate with the Rabbit Reticulate Lysate TnT® system using Luciferase as control. A Luciferase assay was used to determine Luciferase expression levels using a luminometer. The resulting plot is shown in Fig. 2 while taking the standard deviation into account. As can be observed, the difference between the duplicate results is very minimal. The standard deviation varied from 0.10 to 5% leading to very small standard errors. It can further be observed that RTA-PAPS1 has an IC50 at 0.06 nM, slower than RTA IC50 at 0.03 nM but comparable to PAPS IC50 at 0.07 [22, 37, 38]. The IC100 however is attained faster than any of them at 0.24 nM for RTA-PAPS1, twice as fast as RTA IC100 at 0.60 nM. These results show that RTA-PAPS1 is bioactive with a synergetic activity between the RTA and PAPS1 moieties being noticeable.

### Anti-HBV evaluation of recombinant RTA-PAPS1 in HepAD38 cells

**Fig. 3:**
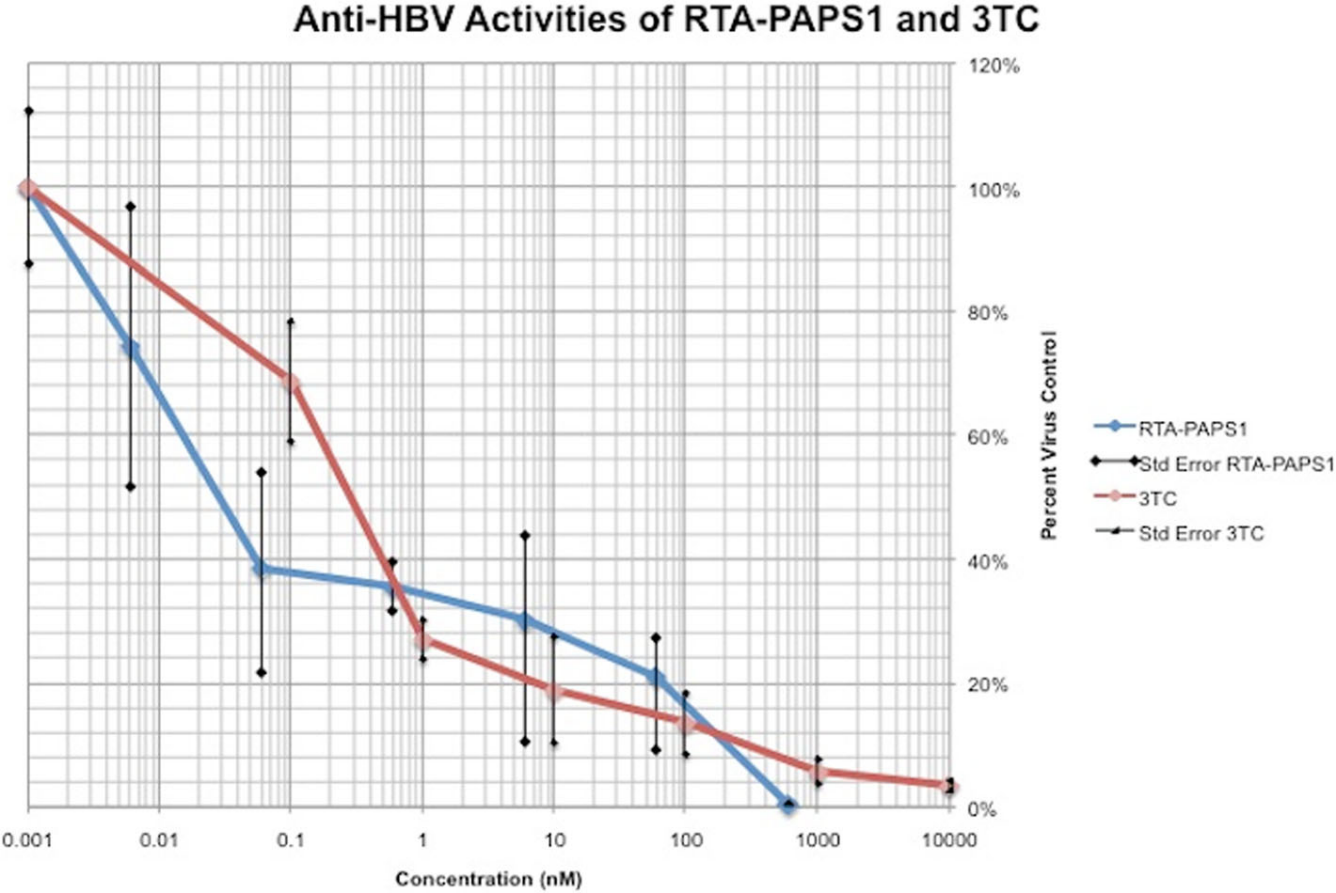
Anti-HBV evaluation of RTA-PAPS1. Recombinant RTA-PAPS1 was tested for its anti-HBV activity using 6 concentrations using a serial dilution by a factor of 10 in growth media (600 nM, 60 nM, 6 nM, 0.6 nM, 0.06 nM, 0.006 nM for RTA-PAPS1 and 10,000 nM, 1000 nM, 100 nM, 10 nM, 1 nM, 0.1 nM for 3TC). Values are calculated as percent of virus DNA control [(amount of virus DNA in treated sample/amount of virus DNA in untreated sample) × 100]. Results represent the mean for three individual experiments

Recombinant RTA-PAPS1 was evaluated for anti-HBV activity and cytotoxicity in the HBV chronically infected cell line AD38 using a six concentrations dose response assay in triplicate. The lamivudine (3TC) control compound was evaluated in parallel. The antiviral efficacy based on quantified DNA copies in the supernatant of both compounds are shown in Fig. 3 in a plot form. RTA-PAPS1 yielded a half-maximal response concentration value (EC50) of 0.03 nM while 3TC yielded an EC50 of 0.3 nM, which is a ten-fold difference. RTA-PAPS1 was not cytotoxic to HepAD38 cells at concentrations up to 600 nM. These results led to a therapeutic index for RTA-PAPS1 of > 21,818, which is a huge improvement over values given in the literature (EC50 of 330 nM and a therapeutic index of 9.3 for PAPS1 alone under comparable conditions on HepG2 2.2.15 cells) [11]. These results clearly show the significant anti-HBV activity of RTA-PAPS1. It is important to note however that the standard deviation between the triplicate reactions ranged from 0.12 to 87% for RTA-PAPS1, and ranged from 0.12 to 46% for 3TC.

### Protein design optimization

**Fig. 4:**
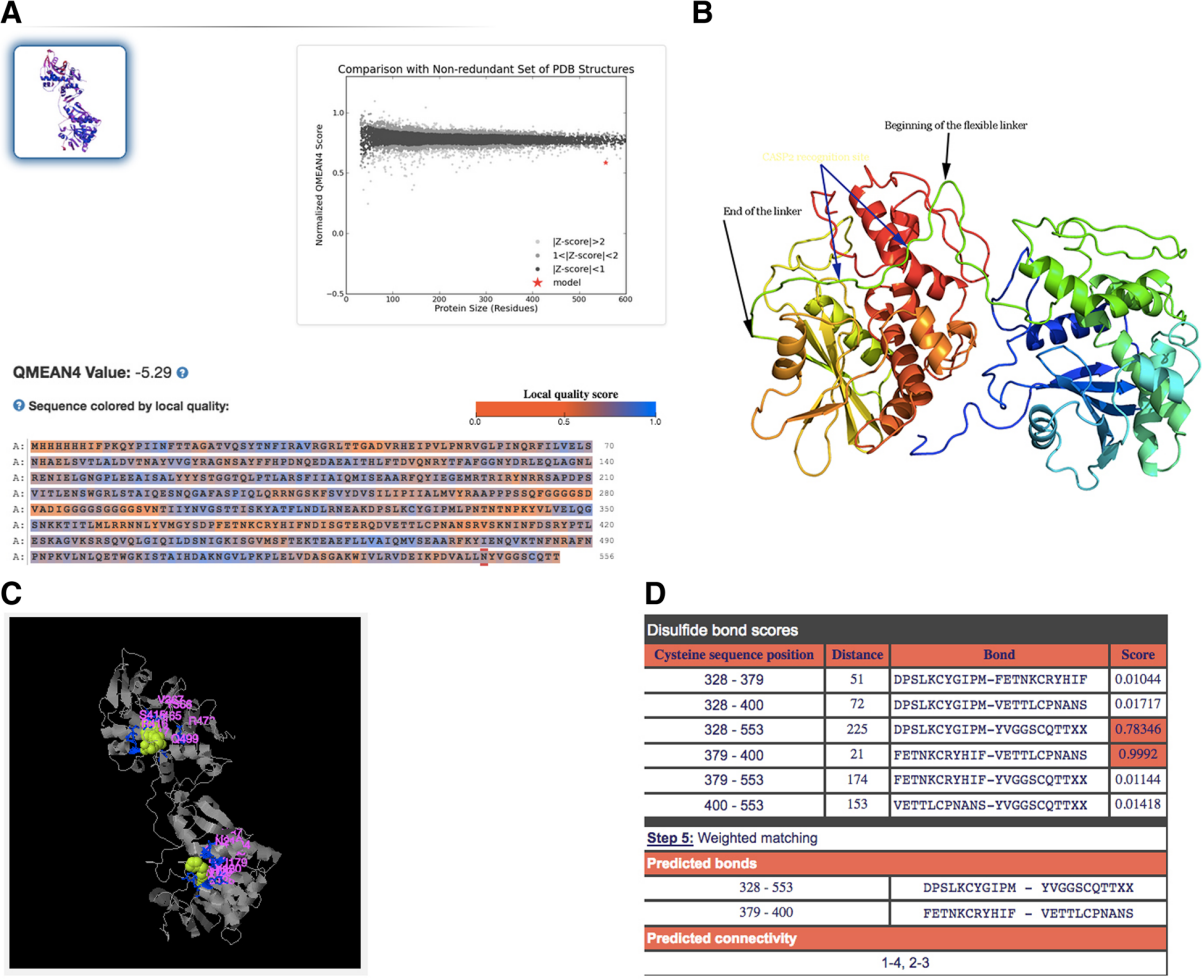
Predicted 3D Protein Structure. **a** QMEAN4 results showing a normalized value > 0.6 with the protein sequence in the bottom and the model in the up left corner. **b** Protein structure as determined by Phyre2 with the black arrows showing the flexible linker at position 275–294 and the blue arrows showing the CASP2 recognition site at position 280–284. Image colored by rainbow (blue to red) N → C terminus. **c** The ligand binding sites of RTAM moiety (up) and of PAP1 moiety (down) as determined by I-Tasser (using the Phyre2 model as one of the templates). **d** The predicted disulfide bonds at 328–553 and 379–400 by DiANNA 1.1 webserver

The design of the recombinant protein RTA-PAPS1 was completely revisited in order to further enhance the effect of the chimeric protein on HBV, reduce general toxicity and increase solubility to improve expression. The resulting design Ricin A Chain Mutant-Pokeweed Antiviral Protein from Leaves (RTAM-PAP1) was run through I-Tasser and Phyre2 and the resulting 3D models validated by Verify 3D. The model generated by Phyre2 passed Verify 3D while the one generated by I-Tasser failed. The one generated by Phyre2 was thus chosen as one of the templates to run I-Tasser again. The newly generated structure by I-Tasser scored higher on Verify 3D than the one generated by Phyre2 and was thus chosen as the model for the other software. The proper disulfide bond formations were confirmed by the DiANNA 1.1 webserver (at positions 328–553 and 379–400). The new model had a normalized QMEAN4 score of > 0.6 and the introduction of the rigid CASP2 recognition site into the flexible linker at position 280–285 insured safe distance between the two proteins to safeguard the function of both moieties and minimize steric hindrance as can be seen in Fig. 4. The grand average of hydropathicity was reduced from − 0.236 for RTA-PAPS1 to − 0.265 for RTAM-PAP1 as was determined by ProtParam, which represents an improvement of 12% in hydrophilicity (results not shown).

### Production and purification of recombinant RTAM-PAP1 in *E. coli* culture

**Fig. 5:**
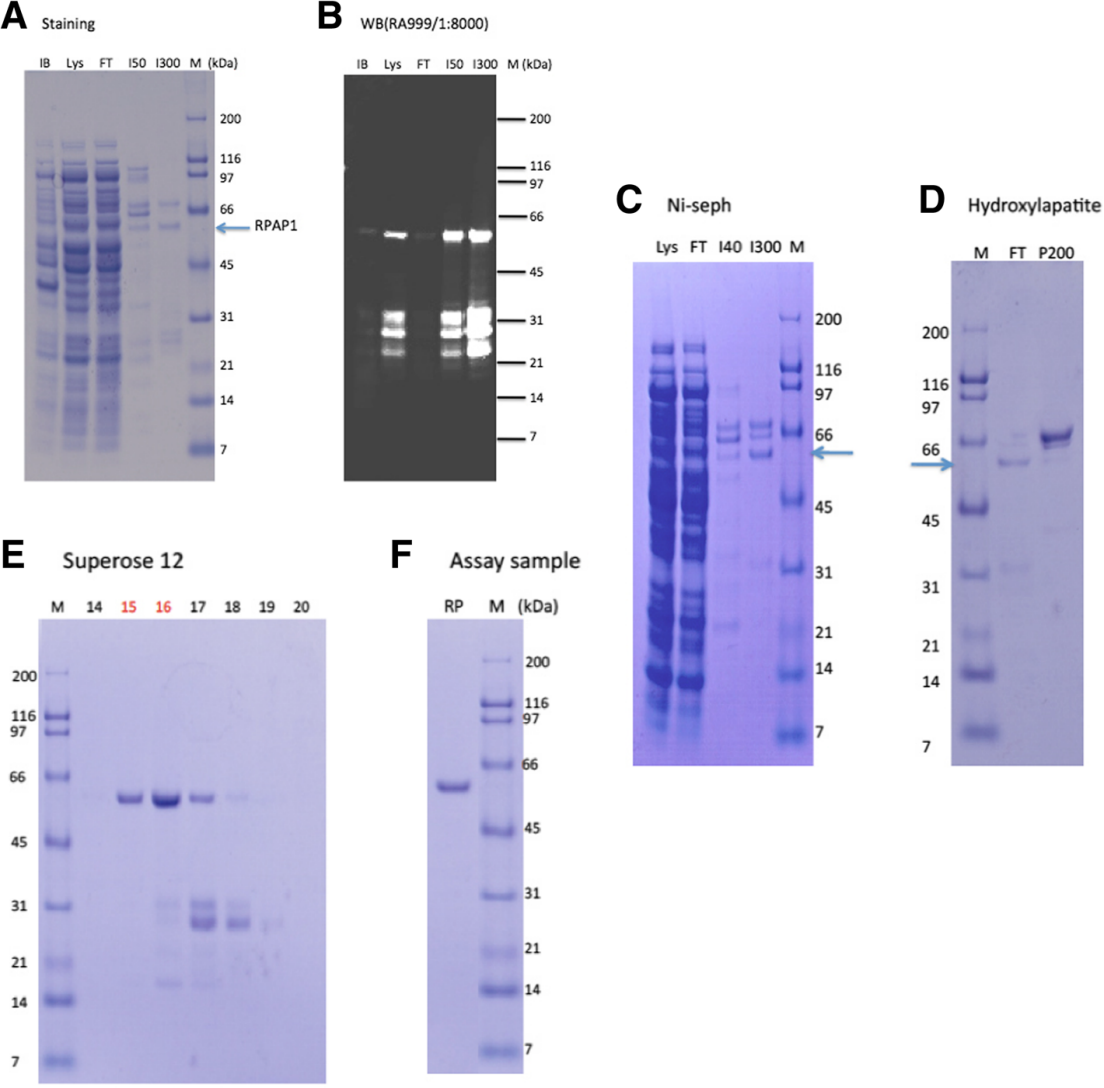
Production and Purification of native RTAM-PAP1. **a** Loosely bound proteins were washed with the lysis buffer containing 50 mM imidazole (I50) on a Ni-sepharose column and RTAM-PAP1 (RPAP1) proteins were then eluted with the elution buffer containing 300 mM Imidazole (I300). **b** The Western Blot using ricin a chain antibody RA999 confirmed the presence of RTAM-PAPS1 at approx. 61.5 kDa. The bands between 21 kDa and 32 kDa are assumed to be degraded or/and premature RTAM-PAP1 proteins. **c** (Lys) from 1 L culture. a) Loosely bound proteins were washed with the lysis buffer containing 40 mM imidazole (I40) on a Ni-sepharose column and RTAM-PAP1 proteins were then eluted with the elution buffer containing 300 mM Imidazole (I300). **d** Co-purified host cell proteins were further separated by a hydroxylapatite column. Most RTAM-PAP1 proteins were retained in the flow through (FT) fraction, while most host cell proteins were bound to the hydroxylapatite column (P200 elution). **e** RTAM-PAP1 was peaked at fraction 15 and 16. The purest fraction (F15) was estimated at > 95% homogeneity (**f**) and was used for the inhibition assay

The production of RTAM-PAP1 was first tested under the same conditions as previously determined for RTA-PAPS1 and resulted in good production of native proteins. Soluble RTAM-PAP1 was recovered from the lysate, purified by Ni-sepharose column and analyzed by SDS-PAGE and Western Blot (Fig. 5a, b). The production from 1 L culture under the same conditions gave equally good results (Fig. 5c.). The purified proteins were then submitted to a second purification step using hydroxylapatite column, which showed good separation of RTAM-PAP1 from co-purified host proteins (Fig. 5d). The degraded (and/or premature) products were further separated by gel filtration on an FPLC column of Superose 12 (Fig. 5e) and the purest fraction (F15) reached > 95% homogeneity at a concentration of 0.1 mg/ml (Fig. 5f) and was used for the protein synthesis inhibition assay.

### Inhibitory activity of recombinant RTAM-PAP1 vs. RTA-PAPS1 in the rabbit reticulate lysate TnT® system

**Fig. 6:**
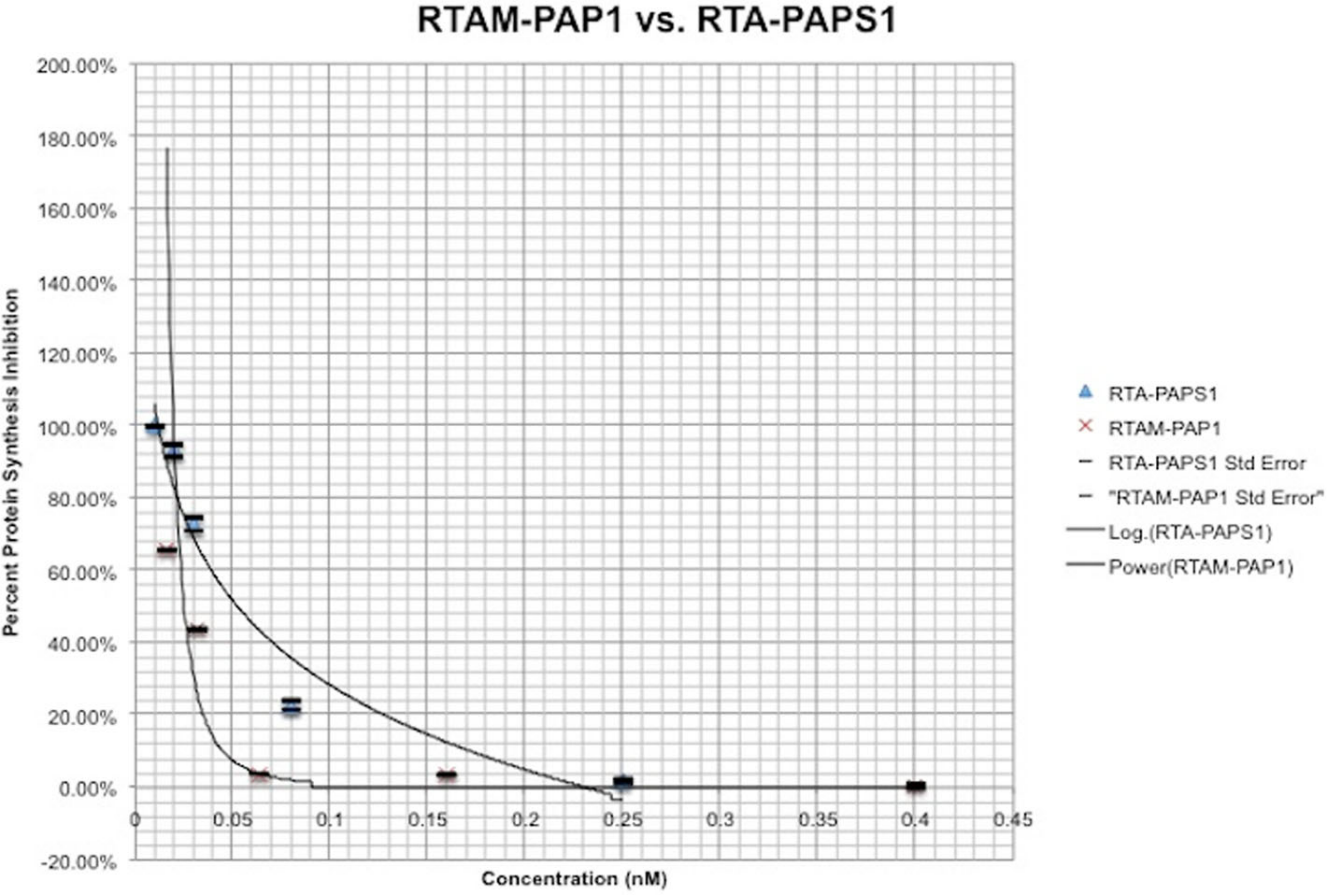
Comparative inhibition activity of RTAM-PAP1 and RTA-PAPS1 in the TnT transcription/translation assay. Five different concentration points (0.01 nM, 0.02 nM, 0.03 nM, 0.08 nM, 0.25 nM for RTA-PAPS1 and 0.02 nM, 0.03 nM, 0.06 nM, 0.16 nM, 0.40 nM for RTAM-PAP1) were examined. Values are calculated as percent Luciferase protein synthesis compared to control. Results represent the mean for two individual experiments and the curve is the logarithmic regression for RTA-PAPS1

The inhibitory activity of RTAM-PAP1 was determined using 5 different concentrations, in duplicate, of purified RTAM-PAP1 on the Rabbit Reticulate Lysate TnT® system using Luciferase as the control as previously described. The resulting comparative plot of the activity on protein synthesis of both fusion proteins is shown in Fig. 6 while taking into account the standard deviations that ranged from 0.1 to 1%. As can be observed, the plot showed minimal difference between duplicates. It also shows that RTAM-PAP1 has an IC50 at 0.03 nM, the same as RTA IC50 at 0.03 nM, which is twice as fast as RTA-PAPS1 IC50 at 0.06 nM and about ten times faster than PAP1 IC50 at 0.29 nM [38]. The IC100 however is attained faster than any of them at 0.09 nM for RTAM-PAP1, which is a bit less than three times faster than RTA-PAPS1 IC100 at 0.24 nM. These results show that RTAM-PAP1 is bioactive, both moieties’ complementary catalytic activities functional, with minimal steric hindrance if any, and with a significant gain of function.

## Discussion

The chimeric protein RTA-PAPS1 was expressed only in inclusion bodies with very little solubility, except under heavy denaturing conditions. The refolding process was successful to a certain extent as more than one conformation was observed. This was probably due to the two free Cysteine residues in RTA and to the nature of the semi-flexible linker, which allowed the close proximity of Cys at position 260 to the Cys residues at position 364 and 385 (confirmed by DiANNA 1.1 webserver and I-Tasser). The addition of TCEP was necessary and a difference in bioactivity (> 2 fold) was observed between samples (results not shown). RTA-PAPS1 with the addition of TCEP was very bioactive and with a noticeable synergetic activity between RTA and PAPS1, which was probably limited by steric hindrance once again due to the nature of the semi-flexible quality of the linker. This was confirmed during the anti-HBV assays. The significant anti-HBV activity of RTA-PAPS1 was apparent and probably due to the ability of both moieties to depurinate rRNA but also polynucleotide, single-stranded DNA, double stranded DNA and mRNA [11, 39]. HBV is a double stranded DNA reverse transcriptase virus. The observed higher standard deviations for RTA-PAPS1 compared to those observed for 3TC between samples at the same concentration during the anti-HBV assay were probably due to the presence of different conformations of the same protein. Additionally, dubious results were seen at the highest concentration of 600 nM for cytotoxicity, which were probably due to an unwanted reaction that occurs when the protein buffer solution to growth media ratio is high (> 30% at 600 nM). Furthermore, it was observed that the steric hindrance diminished the ability of RTA-PAPS1 to penetrate infected cells by > 2 fold compared to PAPS1 alone (the activity of RTA-PAPS1 on other viruses such as HIV-1 for example was found to be much lower than that of PAPS1 alone, results not shown). It was for these reasons that the decision to redesign the fusion protein with PAP1 and a new linker was taken. The decision to engineer an entirely new linker was taken despite the slight possibility that the natural semi-flexible linker used might have been the reason for the significant anti-HBV activity observed. Indeed, the natural semi-flexible linker might have allowed the fusion protein to adopt a unique conformation with a novel or improved existing anti-HBV activity.

The fusion protein RTAM-PAP1 expression went very well as we almost exclusively obtained native protein production with high solubility (barely any in inclusions bodies). It was however necessary to use a three step purification protocol in order to obtain soluble proteins with > 90% homogeneity. Nonetheless, 0.1 mg of protein at > 95% purity and 0.22 mg of protein at > 90% purity were obtained from 1 L of culture. This yield is probably explained by the increased toxicity of PAP1 to *E. coli* compared to that of PAPS1 (> 10 fold) [40]. The bioactivity of RTAM-PAP1 was increased, much more than expected with very little to no sign of steric hindrance. The introduction of the two point mutations in the RTA moiety and of the flexible linker really made a difference in solubility and activity. Also, perhaps, fine-tuning the formulation buffer to better preserve protein integrity allowed for optimum activity. The synergetic effect of both moieties was very apparent and probably due to the fact that RTA and PAP1 do not dock onto the ribosome at the same site and, thus, led to a reduction of partially depurinated and still functional ribosomes [19].

## Conclusion

The chimeric proteins combining RTA and PAPs are potent novel anti-viral proteins with gain of function in protein synthesis inhibition activity and anti-HBV activity in vitro with minimal cytotoxicity*.* The introduction of two point mutations in RTA and of a flexible linker greatly improved solubility and activity. RTAM-PAP1 can be overexpressed, recovered and purified from soluble lysate. It is expected that the anti-viral properties of RTAM-PAP1 against plant and animal pathogens will be even greater than that of either RTA-PAPS1 or PAPs with even lesser general toxicity. Indeed, this should be true as long as the original natural semi-flexible linker used did not have an unforeseen positive effect on function of RTA-PAPS1. For these reasons, it is the opinion of the authors that a full characterization of RTAM-PAP1 activity against chronic HBV infection be done both in vitro and, if successful, in vivo as it is a potential potent new therapeutic that can be produced at low costs to be used as a standalone or in combination with existent therapies.

## Abbreviations

EC50: Half-maximal response concentration of a drug
IC100: 100% protein synthesis inhibitory concentration
IC50: 50% protein synthesis inhibitory concentration
PAP1: Pokeweed antiviral protein isoform 1 from leaves
PAPs: Pokeweed antiviral proteins
PAPS1: Pokeweed antiviral protein isoform 1 from seeds
RTA: Ricin A Chain
